# Immediate postpartum cardiac surgery for critical congenital heart disease: cardiopulmonary bypass management and early outcomes in 21 neonates

**DOI:** 10.3389/fcvm.2026.1750479

**Published:** 2026-04-15

**Authors:** Jialu Wang, Na Miao, Xiaojuan Zhang, Xueting Zhao, Ju Zhao

**Affiliations:** Department of Extracorporeal Circulation and Mechanical Circulatory Support, Center for Cardiac Intensive Care, Beijing Anzhen Hospital Affiliated to Capital Medical University, Beijing, China

**Keywords:** cardiac surgery, cardiopulmonary bypass, critical congenital heart disease, neonate, outcomes

## Abstract

**Objective:**

To summarize our center's experience with cardiopulmonary bypass (CPB) management in 21 neonates with critical congenital heart disease (CCHD) who underwent immediate postpartum cardiac surgery, and to report their early clinical outcomes.

**Methods:**

This retrospective case series included 21 neonates with CCHD who underwent immediate postpartum cardiac surgery at Beijing Anzhen Hospital between December 2022 and July 2024. The timeliness of surgery, key CPB management parameters, and perioperative clinical outcomes were analyzed.

**Results:**

The median time from delivery to arrival in the cardiac operating room was 8.0 min (IQR: 6.0–19.5 min). The mean CPB duration was 178.4 ± 12.3 min, with a median aortic cross-clamping time of 138.0 min (IQR: 76.0–158.5 min). The lowest mean nasopharyngeal temperature during CPB was 30.2 ± 0.2 °C. The volume of red blood cells transfused during CPB was 142.4 ± 2.2 mL, and the mean hematocrit level after modified ultrafiltration was 38.1%±1.1%. All 21 neonates were successfully weaned from CPB without delayed chest closure. The overall survival rate was 95% (20/21), with a median hospital stay of 21.0 days (IQR: 13.5–28.0 days) and median ICU stay of 10.0 days (IQR: 7.5–15.5 days). Major adverse events occurred in three patients (14.3%): two who recovered after reoperation and one who died from multiorgan failure secondary to low cardiac output caused by obstructive hypertrophic cardiomyopathy.

**Conclusion:**

With standardized and refined CPB management strategies, immediate postpartum cardiac surgery is technically feasible in neonates with CCHD and can achieve acceptable early clinical outcomes. This approach may serve as an alternative strategy for centers lacking rapid interventional capabilities.

## 1 Introduction

Neonatal critical congenital heart disease (CCHD) refers to anatomical cardiac malformations that can cause severe symptoms during the neonatal period. Without timely and appropriate intervention, these conditions carry a high risk of early mortality, often necessitating life-saving interventions or surgery within the neonatal period (1, 2). Examples include hypoplastic left heart syndrome, complete transposition of the great arteries with intact ventricular septum, pulmonary atresia with an intact ventricular septum, and total anomalous pulmonary venous return with obstruction. Upon delivery, the fetal circulation rapidly transitions to a pathological state due to the underlying cardiac malformation, commonly progressing to refractory hypoxemia and multiple organ failure within a few hours. To avoid circulatory failure, early diagnosis and treatment of critical CCHD are crucial for the survival and prognosis of the affected children (3). With the development of prenatal diagnostic technology in recent years, more cases of CCHD are being diagnosed during the fetal period. Additionally, advances in cardiac surgery, cardiopulmonary bypass (CPB), and intensive care have markedly improved outcomes following the surgical correction of CCHD in neonates, with early and intermediate-term survival rates now exceeding 90% (4). To further improve the success rate of surgical treatment for critical CCHD, our center pioneered the concept of immediate postpartum surgical correction of critical CCHD. Immediate postpartum surgical correction of CCHD is achieved via a multidisciplinary approach that performs cardiac surgery immediately after delivery to correct cardiac anomalies within the extremely short fetal circulatory window and promptly restore normal physiological status. The main goals of this approach are: (1) to correct the structural abnormalities as early as possible, thereby reducing complications such as severe hypoxia, heart failure, and multiple organ failure; (2) to utilize the regenerative potential of the neonatal myocardium to promote postoperative recovery (5); (3) to avoid further structural and morphological alterations of the heart and pulmonary vessels caused by the deformities, such as myocardial hypertrophy and delayed pulmonary vascular development.

Herein we present the results of a retrospective analysis of the clinical data and postoperative clinical outcomes of 21 neonates with CCHD who underwent immediate postpartum cardiac surgery at Beijing Anzhen Hospital from December 2022 to July 2025. This summary of our center's experience in CPB management of these patients provides insight for improving treatment outcomes for neonates with CCHD.

## 2 Materials and methods

### 2.1 Patients

This retrospective study included neonates who were prenatally diagnosed with CCHD and underwent immediate postpartum cardiac surgery at Beijing Anzhen Hospital between December 2022 and July 2025. The inclusion criteria were: (1) CCHD confirmed by fetal ultrasound with dependence on the ductus arteriosus for survival; (2) gestational age at birth ≥36 weeks; and (3) birth weight ≥2.0 kg. Patients were excluded if they had severe intrauterine distress (Apgar score <7) or any comorbid genetic syndrome. This study was approved by the Ethics Committee of Beijing Anzhen Hospital, Capital Medical University (No. 2025201x). Due to the retrospective nature of the study, the requirement for informed consent from patients' guardians was waived.

### 2.2 Clinical data collection

Perioperative data from birth to discharge were collected from the electronic medical records system, including: preoperative data (demographic characteristics, prenatal diagnosis, echocardiographic findings, and laboratory results); intraoperative data [surgical procedure, CPB duration, aortic cross-clamping time, cerebral near-infrared spectroscopy (NIRS) values, blood lactate level, and mixed venous oxygen saturation]; and postoperative data [duration of mechanical ventilation, duration of intensive care unit (ICU) stay, length of hospital stay, mortality, blood gas analysis results, and major adverse events].

### 2.3 Preoperative preparation

The immediate postpartum cardiac surgery treatment team at our center is composed of multidisciplinary experts with extensive experience in the diagnosis and treatment of CCHD, including cardiologists and surgeons, cardiac sonographers, extracorporeal circulation physicians, anesthesiologists, intensive care physicians, and nursing teams. In our center, the cesarean section operating room and the cardiac operating room are connected by a dedicated passageway. The CPB circuit is kept primed and maintained at a constant temperature of 35 °C on standby. Additionally, one unit of O-negative washed red blood cells is kept on standby.

### 2.4 Anesthesia and rapid blood typing

Each neonate was admitted to the operating room, intubated, mechanically ventilated, and administered combined intravenous-inhalation anesthesia. Femoral arterial pressure monitoring was performed, accompanied by the establishment of an internal jugular venous access. Near-infrared spectroscopy (NIRS) was employed to monitor regional oxygen saturation. For cerebral oxygen monitoring, the sensor patches were placed symmetrically approximately 1 cm above the supraorbital ridge, centered along the midline. For renal regional oxygen saturation monitoring, the patches were positioned bilaterally along the spine, below the costal margin. Immediately after delivery, umbilical cord blood was drawn for rapid blood typing. After induction of anesthesia, blood was drawn from the femoral artery for verification of type and cross-matching. If initial and confirmatory blood typing was inconclusive, cross-matched O-negative washed red blood cells were used.

### 2.5 Extracorporeal circulation management

The following components were routinely used for the CPB circuit: a Terumo FX05 membrane oxygenator, neonatal tubing pack, cardioplegia delivery system, BLS803 infant ultrafilter, vacuum-assisted venous drainage (VAVD) device, sevoflurane vaporizer, Solin autologous blood recovery system, an 8 Fr DLP arterial cannula, a 10 Fr right-angle venous cannula for the superior vena cava, and a 14 Fr straight-tip venous cannula for the inferior vena cava.

The CPB priming solution consisted of 90 mL of washed red blood cells, 40 mL of 20% human albumin, 85 mL of balanced electrolyte solution, 15 mL of 5% sodium bicarbonate, and 20 mg of heparin. The total priming volume was approximately 230 mL, and the venous reservoir level was maintained at approximately 50 mL.

For anticoagulation, an initial heparin dose of 3 mg/kg was administered intravenously after anesthesia induction. During CPB, the activated clotting time (ACT) was measured every 30 min and maintained at >480 s. If the ACT fell below 400 s, an additional 1 mg/kg of heparin was administered. After weaning from CPB, heparin was neutralized with protamine at a 1:1 ratio to the initial heparin dose, administered by slow infusion.

The myocardial protection strategy was as follows: after establishing full-flow CPB, cooling was initiated. Once the nasopharyngeal temperature fell below 32 °C, the ascending aorta was clamped, and cold HTK (histidine-tryptophan-ketoglutarate) cardioplegic solution was administered via the aortic root at a dose of 50–60 mL/kg over 5–6 min. An ice/water mixture was positioned over the cardiac surface to maintain local hypothermia.

Target CPB parameters included a perfusion flow rate of 120–150 mL/kg/min, a mean arterial pressure (MAP) of 20–50 mmHg, venous oxygen saturation (SvO₂) > 70%, and a hematocrit (HCT) of 25%–28%. During rewarming, 10 mL of 20% human albumin and 60 mL of washed red blood cells were added to the circuit. The HCT was maintained above 35% following modified ultrafiltration.

Intraoperative ultrafiltration was performed according to the degree of hemodilution to maintain the target HCT level. Zero-balance ultrafiltration (ZBUF) was initiated after rewarming, with a target exchange volume of 100–120 mL/kg. Modified ultrafiltration (MUF) was performed for 8–10 min after weaning from CPB.

The following monitoring methods were applied for organ protection during CPB. Cerebral oxygen saturation (rSO₂) was continuously monitored by NIRS, with the goal of maintaining rSO₂ > 50% or a decrease of <20% from baseline. Renal protection was ensured by monitoring renal oxygen saturation combined with urine output assessment. To attenuate the inflammatory response, methylprednisolone (30 mg/kg) was administered immediately after CPB initiation.

### 2.6 Observed outcomes

The primary outcome measure in this study was the postoperative occurrence of a major adverse event (MAE), defined as at least one of the following conditions: cardiac arrest, need for reoperation, use of advanced life support systems, or death. Secondary outcomes included the duration of postoperative mechanical ventilation (prolonged mechanical ventilation was defined as cumulative postoperative mechanical ventilation duration >7 days), length of ICU stay, need for peritoneal dialysis, need for reintubation, length of hospital stay, and hospitalization costs.

### 2.7 Statistical methods

This was a retrospective descriptive case series study. All data analyses were performed using SPSS 27.0 statistical software. Quantitative data that followed a normal distribution are expressed as mean ± standard deviation (SD). Non-normally distributed quantitative data are expressed as median [interquartile range (IQR)]. Categorical data are expressed as frequencies and percentages.

## 3 Results

### 3.1 Patient characteristics

Between December 2022 and July 2025, 21 neonates with CCHD underwent immediate postpartum cardiac surgery at our center. The cohort included 10 females (47.6%) and 11 males (52.4%). All 21 patients were ductus-dependent, with diagnoses including transposition of the great arteries with an intact ventricular septum in 7 cases, transposition of the great arteries with a restrictive ventricular septal defect in 4 cases, pulmonary atresia with an intact ventricular septum in 4 cases, severe pulmonary stenosis in 3 cases, obstructive total anomalous pulmonary venous connection in 1 case, severe aortic stenosis in 1 case, and anomalous origin of the pulmonary artery in 1 case. Gestational age at birth ranged from 37 to 39 weeks, and the mean birth weight was 3.2 kg (range 2.8–3.4 kg) (Table 1).

**Table 1:** Clinical characteristics of the cCHD patients.

Patient #	Gender	Gestational age (weeks)	Birth weight (kg)	Time from delivery to ventilator (min)	Operation time (h)	Diagnosis	Surgery type	Outcome
1	Male	38	3.4	17	3.1	Obstructed TAPVC	TAPVC repair	Recovered and was discharged from hospital
2	Male	39	3.3	22	5.4	TGA/IVS	ASO	Recovered and was discharged from hospital
3	Female	38	3.7	12	6.4	TGA/IVS	ASO	Recovered and was discharged from hospital
4	Male	38	3.4	8	5.4	AOPA	AOPA repair	Second surgery
5	Male	38	3.4	6	5.5	TGA/VSD	ASO + VSD repair	Recovered and was discharged from hospital
6	Female	38	4.4	5	3.4	S-PS	Pulmonary valvuloplasty	Recovered and was discharged from hospital
7	Female	39	5.0	10	3.4	TGA/IVS/HCM	ASO	Died
8	Male	38	2.7	7	4.1	PA/IVS	PA repair + RVOT reconstruction	Recovered and was discharged from hospital
9	Female	37	3.1	8	3.9	S-AS	Aortic valvuloplasty	Recovered and was discharged from hospital
10	Female	37	2.3	7	3.9	PA/IVS	PA repair + RVOT reconstruction	Recovered and was discharged from hospital
11	Female	37	3.1	6	3.2	PA/IVS	PA repair + RVOT reconstruction	Recovered and was discharged from hospital
12	Male	38	2.8	6	3.7	S-PS	Pulmonary valvuloplasty + RVOT reconstruction	Second surgery
13	Male	39	3.8	129	5.2	TGA/IVS	ASO	Recovered and was discharged from hospital
14	Female	37	2.4	34	8	TGA/IVS	ASO	Recovered and was discharged from hospital
15	Female	37	2.8	24	4.4	PA/IVS	PA repair + RVOT reconstruction	Recovered and was discharged from hospital
16	Male	38	2.7	30	5.9	TGA/IVS	ASO	Recovered and was discharged from hospital
17	Female	37	3.3	7	6.5	TGA/IVS	ASO	Recovered and was discharged from hospital
18	Male	38	3.2	16	6	TGA/VSD	ASO + VSD repair	Recovered and was discharged from hospital
19	Female	37	2.9	2	3.7	S-PS	Pulmonary valvuloplasty	Recovered and was discharged from hospital
20	Male	38	3.0	10	5.5	TGA/VSD	ASO + VSD repair	Recovered and was discharged from hospital
21	Male	38	3.4	5	5.2	TGA/IVS	ASO	Recovered and was discharged from hospital

TAPVC, total anomalous pulmonary venous connection; TGA/VSD, transposition of the great arteries/ventricular septal defect; TGA/IVS, transposition of the great arteries/intact ventricular septum; S-PS, severe pulmonary stenosis; PA/IVS, pulmonary atresia/intact ventricular septum; S-AS, severe aortic stenosis; AOPA, anomalous origin of the pulmonary artery; ASO, arterial switch operation; RVOT, right ventricular outflow tract; HCM, hypertrophic cardiomyopathy.

### 3.2 CPB-related parameters and clinical outcomes

All 21 infants were successfully placed on CPB after delivery. Ten infants received intraoperative transfusions of O-negative washed red blood cells due to cross-matching incompatibility; no adverse reactions were observed. The detailed data related to CPB as well as the occurrence of different outcomes are presented in Table 2. The median time from delivery to arrival in the operating room was 8.0 min (IQR: 6.0–19.5 min). A total of 3 patients (14.3%) experienced MAEs in this study, including 1 patient who died from multi-organ failure after implementation of emergency extracorporeal membrane oxygenation (ECMO) support and 2 patients who underwent reoperation. No cases of delayed chest closure were reported. Postoperatively, 10 patients required peritoneal dialysis, two required reintubation, and four (19.1%) required prolonged mechanical ventilation, with a median duration of 93.0 h (IQR: 47.8–117.0 h). Additionally, all infants underwent routine cranial ultrasound screening during hospitalization, and no severe intracranial hemorrhage or ischemic lesions requiring intervention were identified.

**Table 2:** CPB-related data and postoperative complications among cCHD patients.

Operative data and postoperative morbidity	n (%)/median (IQR)/mean ± SD
Surgical data	
Surgical duration (h)	4.9 ± 0.3
CPB duration (min)	178.4 ± 12.3
Aortic cross-clamping time (min)	138.0 (76.0–158.5)
Total heparin dose during CPB (mg)	35.0 (30.0–40.0)
Minimum nasopharyngeal temperature during CPB ( °C)	30.2 ± 0.2
Blood transfusion volume during CPB (mL)	142.4 ± 2.2
HCT after modified ultrafiltration (%)	38.1 ± 1.1
Postoperative complications	
Major adverse events [n (%)]	3 (14.3%)
Death [n (%)]	1 (4.8%)
Reoperation [n (%)]	2 (9.5%)
Re-intubation [n (%)]	2 (9.5%)
Peritoneal dialysis [n (%)]	10 (47.6%)
Mechanical ventilation duration (h)	93.0 (47.8–117.0)
Prolonged mechanical ventilation [n (%)]	4 (19.1%)
ICU length of stay (d)	10.0 (7.5–15.5)
Hospital length of stay (d)	21.0 (13.5–28.0)
Hospitalization costs (¥10,000)	17.4 (12.4–22.0)

IQR, interquartile range; SD, standard deviation; CPB, cardiopulmonary bypass; HCT, hematocrit.

Twenty (95%) infants survived and were discharged from the hospital, and one died in the hospital. In the single non-surviving case, immediate postnatal echocardiography confirmed TGA/IVS with fusiform hypertrophy of the mid-ventricular septum. Left ventricular outflow tract (LVOT) blood flow velocity was normal with no evidence of obstruction. The surgical indications were clear, and the patient underwent arterial switch operation (ASO), ductus arteriosus ligation, and foramen ovale repair. The initial postoperative recovery was uneventful, and extubation was performed on postoperative day 6. Following extubation, the patient developed hemodynamic instability with a progressively increasing LVOT pressure gradient. The progressive septal hypertrophy severely compromised cardiac function, necessitating reintubation. On postoperative day 17, the patient developed circulatory failure and was placed on ECMO support. Despite six days of ECMO support, the patient died after ECMO withdrawal due to progressive anasarca, anuria, hyperlactatemia, coagulopathy, and electrolyte imbalance.

Two patients (9.5%) required reoperation during the same hospital admission. The first patient was diagnosed postnatally by immediate echocardiography with anomalous origin of the left pulmonary artery from the ascending aorta, ventricular septal defect, atrial septal defect, and bicuspid aortic valve. Emergency surgical correction was performed, including repair of the anomalous pulmonary artery, ventricular septal defect closure, atrial septal defect closure, and ligation with division of the patent ductus arteriosus. Although extubated on postoperative day 7, the patient required reintubation due to subsequent pulmonary edema and hypoxemia. Follow-up echocardiography revealed progressively worsening moderate-to-severe tricuspid regurgitation, leading to a tricuspid valvuloplasty 45 days after the initial surgery. The patient recovered well thereafter and was discharged. The second patient was diagnosed immediately after birth with critical pulmonary valve stenosis, bordering on atresia, via echocardiography. This patient underwent emergency surgery comprising pulmonary valvuloplasty, atrial septal defect closure, right ventricular outflow tract augmentation, tricuspid valvuloplasty, and partial patent ductus arteriosus ligation (maintaining a residual lumen of approximately 4 mm). Postoperative course in the ICU was complicated by elevated right ventricular preload and hemodynamic instability, necessitating emergency re-exploration and surgical atrial septostomy. After delayed sternal closure, the patient developed persistent hypoxemia and elevated lactate levels, which prompted a third emergency thoracotomy for further partial patent ductus arteriosus ligation (reducing the residual lumen to approximately 2 mm). The patient was successfully extubated on postoperative day 8, showed steady improvement, and was discharged on postoperative day 33.

## 4 Discussion

Although the mortality rate among infants with CHD has decreased, CHD remains the leading cause of death attributed to birth defects, accounting for 30%–50% of such deaths, and significantly affects the quality of life and life expectancy of children who do survive (6). For CCHD that results in dependence on fetal circulation, conservative treatment is usually ineffective; once diagnosed, these cases require urgent or emergency surgery. At present, advances in surgical and perioperative management techniques have enabled safe completion of neonatal cardiac surgery. To further improve outcomes for CCHD, our center pioneered the concept of “immediate postpartum cardiac surgery,” in which cardiac anomalies are corrected within the brief window after delivery before clinical deterioration occurs.

The immediate postpartum cardiac surgical repair strategy reported in this study differs significantly from the current mainstream “pharmacological/interventional stabilization-elective surgery” paradigm. The rationale for this paradigm shift stems from the specific clinical context and resource conditions at our center. Prior to initiating this study, our center experienced two cases of prenatally diagnosed transposition of the great arteries with intact ventricular septum. Despite immediate initiation of prostaglandin E1 therapy after birth, both neonates lost the opportunity for surgery within hours due to progressive restriction of ductal patency. This experience highlighted that, for duct-dependent congenital heart disease, the traditional “stabilization period” itself may represent a high-risk window. Additionally, our center does not currently perform routine neonatal interventional balloon atrial septostomy. Therefore, for neonates who depend on atrial-level shunting to maintain oxygenation, once the interatrial communication becomes restrictive, we lack rapid and effective bedside interventions. Against this backdrop, advancing surgical timing to the immediate postpartum period essentially represents a risk redistribution strategy—substituting planned, controllable surgical risks for uncontrollable, potentially sudden preoperative deterioration risks. Its core value lies in demonstrating that, with the support of a comprehensive multidisciplinary team system and standardized cardiopulmonary bypass management, this strategy is technically feasible and yields acceptable early clinical outcomes. Widespread adoption of this strategy should be strictly limited to centers with comprehensive multidisciplinary teams encompassing prenatal diagnosis, obstetrics, cardiac surgery, cardiopulmonary bypass, and intensive care, and requires careful weighing of its potential benefits and risks.

After years of development and refinement, our center has established standardized, integrated prenatal and postpartum treatment processes toward the goal of achieving seamless integration of obstetrics and cardiac surgical teams. For children born in extremely critical condition who require immediate cardiac surgery after birth, elective cesarean section is the preferred delivery method. After delivery, routine care and Apgar scoring are performed rapidly, and the neonate is transferred to the adjacent cardiac operating room where immediate transthoracic echocardiography is performed to confirm the diagnosis. Anesthesia induction, orotracheal intubation, and arterial and venous cannulation are performed simultaneously. Blood is drawn for blood typing and cross-matching to expedite preoperative preparation. Neonatal cross-matching is particularly challenging due to the high incidence of hemolytic disease in the neonatal period. The presence of maternal IgG antibodies can lead to the development of immune antibodies in the neonate, resulting in cross-matching incompatibility. According to pediatric transfusion guidelines, O-negative washed red blood cells may be used for neonates requiring emergency transfusion when blood type is uncertain (7). In the present study, 10 neonates with cross-matching incompatibility received O-negative washed red blood cells during CPB, with no adverse transfusion reactions.

As a perfusionist-led study, the core contribution of this report lies in systematically summarizing the management experience of cardiopulmonary bypass implemented at the unique time point of the immediate postpartum period. Although the CPB management strategies employed generally follow conventional neonatal cardiac surgery principles (e.g., miniaturized circuitry, modified ultrafiltration, HTK cardioplegia), immediate postpartum surgery presents unique physiological challenges: hemodynamic fluctuations during the transition from fetal to neonatal circulation, acute volume shifts following placental separation, and heightened organ protection demands associated with ultra-early intervention. To address these challenges, our center prioritized the following strategies: (1) use of high-performance miniaturized oxygenators and circuits with vacuum-assisted venous drainage to minimize priming volume; (2) implementation of refined intraoperative monitoring (e.g., blood flow, temperature, hemodynamics, blood gases) to maintain circulatory stability; (3) combined ultrafiltration and blood salvage to maintain HCT at 25%–28% during CPB, increasing to >35% after modified ultrafiltration; and (4) use of HTK cardioplegic solution ensuring adequate perfusion time (≥5 min) and dose (>50 mL/kg). The integrated application of these strategies provides technical support for the safe performance of immediate postpartum surgery.

This retrospective analysis of 21 neonates with critical congenital heart disease who underwent immediate postpartum cardiac surgery represents the first systematic evaluation of this novel treatment strategy. Regarding clinical outcomes, 20 of the 21 patients (95%) were successfully discharged, indicating favorable short-term survival benefits. The median total hospital stay was 21.0 days (IQR: 13.5–28.0 days), and the median ICU stay was 10.0 days (IQR: 7.5–15.5 days). Compared with data from two recent domestic studies on neonatal complex congenital heart disease surgery—which reported mean total hospital stays of approximately 18 ± 9 days and median ICU stays of approximately 14 days—the findings of this study are highly comparable, with a trend toward shorter ICU stay. Notably, our cohort consisted of prenatally diagnosed neonates, theoretically a higher-risk subgroup for immediate surgery. Despite potentially more severe baseline conditions, this strategy achieved intensive care efficiency comparable to, or even better than, that of conventional timing surgery, providing preliminary support for its feasibility in high-risk neonatal populations. Regarding safety, the major adverse event rate associated with immediate postpartum surgery was 14.3% (3/21), including one in-hospital death following emergency ECMO support and two reoperations, with the overall complication rate remaining within an acceptable range. The clinical benefits of this strategy may be attributed to intervention within the physiological window of fetal circulation, during which the heart retains certain compensatory characteristics and has not yet sustained irreversible organ damage—conditions that create a uniquely favorable physiological environment for anatomical correction.

The present study has several limitations. First, as a single-center retrospective case series with a small sample size (*n* = 21), its statistical power and generalizability are limited. Second, the study focused primarily on summarizing cardiopulmonary bypass management; therefore, data collection emphasized intraoperative parameters and basic outcomes. Detailed postoperative ICU data—including hemodynamic changes, daily inotropic scores, and transfusion details—were not systematically collected, limiting analysis of early postoperative recovery. Third, long-term neurodevelopmental follow-up is lacking, a critical endpoint for this intervention. We have initiated a prospective follow-up study for this cohort, and results will be reported separately. Fourth, the absence of a concurrent control group undergoing surgery at conventional timing precludes direct comparison between the two strategies. Future multicenter prospective studies are needed to further validate this approach.

## 5 Conclusion

This retrospective analysis of 21 neonates with CCHD undergoing immediate postpartum cardiac surgery demonstrates that, with standardized and refined CPB management, this strategy is technically feasible in centers with comprehensive multidisciplinary teams. The overall survival rate was 95%, with acceptable early clinical outcomes. Under specific resource conditions, this approach may serve as an alternative to the conventional “stabilization-elective surgery” paradigm, substituting planned surgical risks for unpredictable preoperative deterioration. However, generalization of these findings requires caution. Future multicenter prospective studies with long-term neurodevelopmental follow-up are warranted to further validate the efficacy and safety of this strategy.

## Data Availability

The original contributions presented in the study are included in the article/supplementary material, further inquiries can be directed to the corresponding author.
